# THE ASSOCIATION BETWEEN CLOSE RELATIONSHIPS AND HAPPINESS AMONG OLDER ADULTS

**DOI:** 10.1093/geroni/igae098.4384

**Published:** 2024-12-31

**Authors:** Elnaz Abaei, Peter Martin

**Affiliations:** Iowa State University, Ames, Iowa, United States; Iowa State University, Ames, Iowa, United States

## Abstract

Close relationships have long been recognized as crucial in shaping older adults’ happiness and well-being. This study investigated the association between close relationships (including those with a spouse, children, and friends) and happiness among older adults using random intercept cross-lagged panel models (RI-CLPMs). We pooled waves 11-12, 13-14, and 15-16 from the RAND file of the Health and Retirement Study (HRS). Our analytic sample included 15,758 individuals aged 50 years and older (Mean age=67.14). We ran three RI-CLPMs to examine how close relationships with spouse, children, and friends affect the happiness of older adults. All models demonstrated a good fit with the data (spouse’s model, χ2 (df=7)=6.979, p=.43, CFI=TLI=1.00; children’s model, χ2 (df=8)=13.91, p=.08, CFI=TLI=1.00; friend’s model, χ2 (df=8)=17.35, p=.03, CFI=.999, TLI=.996). We also conducted a sensitivity analysis by computing models without missing data imputation. The results were mostly consistent with the initial analysis. In the initial waves, we did not find carry-over and spill-over effects between close relationships and happiness. However, carry-over effects were observed for models in the later waves. We also found that married individuals tend to be happier as they age. Additionally, women were more likely to have close relationships with children and friends compared to men. Practical implications should focus on fostering positive interactions among older adults with close social contacts, like encouraging companionship and supportive communication from spouses and children. Group interventions, such as peer support groups, can enhance happiness by monitoring social interactions to promote well-being in older adults.

